# The Role of Dietary Sugars and *De novo* Lipogenesis in Non-Alcoholic Fatty Liver Disease

**DOI:** 10.3390/nu6125679

**Published:** 2014-12-10

**Authors:** J. Bernadette Moore, Pippa J. Gunn, Barbara A. Fielding

**Affiliations:** Department of Nutritional Sciences, Faculty of Health and Medical Sciences, University of Surrey, Guildford, Surrey GU2 7XH, UK; E-Mails: p.gunn@surrey.ac.uk (P.J.G.); B.Fielding@surrey.ac.uk (B.A.F.)

**Keywords:** non-alcoholic fatty liver disease, sugar, fructose, glucose, *de novo* lipogenesis

## Abstract

Dietary sugar consumption, in particular sugar-sweetened beverages and the monosaccharide fructose, has been linked to the incidence and severity of non-alcoholic fatty liver disease (NAFLD). Intervention studies in both animals and humans have shown large doses of fructose to be particularly lipogenic. While fructose does stimulate *de novo* lipogenesis (DNL), stable isotope tracer studies in humans demonstrate quantitatively that the lipogenic effect of fructose is not mediated exclusively by its provision of excess substrates for DNL. The deleterious metabolic effects of high fructose loads appear to be a consequence of altered transcriptional regulatory networks impacting intracellular macronutrient metabolism and altering signaling and inflammatory processes. Uric acid generated by fructose metabolism may also contribute to or exacerbate these effects. Here we review data from human and animal intervention and stable isotope tracer studies relevant to the role of dietary sugars on NAFLD development and progression, in the context of typical sugar consumption patterns and dietary recommendations worldwide. We conclude that the use of hypercaloric, supra-physiological doses in intervention trials has been a major confounding factor and whether or not dietary sugars, including fructose, at typically consumed population levels, effect hepatic lipogenesis and NAFLD pathogenesis in humans independently of excess energy remains unresolved.

## 1. Introduction

Non-alcoholic fatty liver disease (NAFLD) is defined by the accumulation of fat in the liver in the absence of excess alcohol consumption. The incidence of NAFLD worldwide has risen markedly in recent years in parallel with the increasing prevalence of global obesity and recent estimates are that there are approximately one billion cases worldwide [1]. Obesity and type 2 diabetes are strong risk factors for the development of NAFLD [2,3], and it is often regarded as the hepatic manifestation of the metabolic syndrome [4]. However, these interrelationships are complex and clearly dependent on genetic, ethnic and environmental factors, as NAFLD patients may be normal weight [5] and individuals with insulin resistance do not always develop NAFLD [6]. There is no licensed pharmaceutical therapy for the treatment of NAFLD, therefore clinical guidelines to date agree that weight loss through lifestyle modification is the first-line approach along with management of co-morbidities as necessary [7,8]. A number of dietary factors have been implicated in the pathogenesis of NAFLD with a recent focus on dietary carbohydrates, sugar-sweetened beverages and the monosaccharide fructose in particular [9,10,11]. This follows on from older observations of carbohydrate-induced hypertriglyceridemia [12]. Fructose has been scrutinized in part because its hepatic metabolism differs from glucose and high fructose intakes have been shown to alter hepatic insulin sensitivity, increase lipogenesis and ectopic lipid disposition in human [13,14,15] as well as rodent studies [16,17]. Mechanistically, the adverse metabolic effects of high fructose intakes have been linked to its provision of increased substrate to *de novo* lipogenesis (DNL), transcriptional activation of lipogenesis, lipotoxicity and the generation of excess uric acid leading to mitochondrial and endoplasmic reticulum oxidative stress [18,19,20,21]. However, two very recent meta-analyses have concluded that fructose intervention trials examining liver outcomes have largely been done in healthy male participants receiving very high (100–200 g/day) doses of fructose and have often been confounded by excess energy intakes [22,23]. Provocatively, Chung *et al.* [23] suggest there is some evidence that hypercaloric fructose and glucose diets have similar effects on liver fat. 

Despite a large body of work the health and metabolic effects of fructose and other dietary sugars are unresolved [24]. Whether or not fructose affects hepatic lipogenesis and NAFLD pathogenesis independently of excess energy remains an outstanding question. Here we review data from human and animal intervention and stable isotope tracer studies relevant to the role of dietary sugars on NAFLD development and progression, in the context of typical sugar consumption patterns and dietary recommendations worldwide.

## 2. Dietary Sugars and Health

### 2.1. Public Health Recommendations

The role of dietary sugars in health and disease has long been contentious. While high intakes have been associated with increased risk for obesity, cardiovascular disease, diabetes and dental caries in addition to NAFLD, existing data are often ambiguous [25,26]. Public health advice regarding dietary carbohydrate and sugar intake varies depending on the advising body [27]. Both the World Health Organization (WHO) and the UK Scientific Committee on Nutrition (SACN) have commissioned reviews on dietary carbohydrates and have released draft reports for public consultation on guidelines for sugar consumption this year (2014) [28,29]. One benefit of these reviews has been the clarification of the definitions of various groups of sugars including “free sugars”, which are now defined as: all monosaccharides (glucose, fructose, galactose) and disaccharides (sucrose, lactose, maltose, trehalose) added to foods by the manufacturer, cook, or consumer. The sugars naturally present in honey, syrups, and fruit juices are also included in the definition [30].

In advance of updating their guidelines, the WHO commissioned systematic reviews on dietary sugars and body weight and dental caries [30,31]. The review of dietary sugar and body weight examined over 1700 research trials and cohort studies and concluded that, in adults consuming *ad libitum* diets, the intake of free sugars was a determinant of body weight [30]. The results showed that reduced intake of sugars was associated with a decrease in body weight (95% CI 0.39 to 1.21 kg), and increased intake of free sugars was associated with a corresponding increase in body weight (0.30 to 1.19 kg). However, when sugars were exchanged isoenergetically with other carbohydrates there was no effect. The authors acknowledged the significant heterogeneity evident among the trials included as, in part, inherent to interventions in free living people consuming *ad libitum* diets, but conclude that their results give an indication of what might be achieved by population changes in intake of dietary sugars. Interestingly, the draft recommendations of the WHO, for reduced intake of free sugars throughout the life-course and the intake of free sugars not to exceed 10% of total energy, were based on the evidence for reducing the risk of dental caries rather than obesity [28]. 

Separately, SACN also commissioned systematic reviews to inform their report and concluded there is a dose response relationship between total energy intake and % of energy from sugars and that “in relation to both improving oral health and reducing the risk of weight gain, sugars should provide no more than 10% of dietary energy” [29]. Furthermore, they recommended the dietary reference value for free sugars be set at a population average of around 5% of dietary energy in order to achieve no more than 10% of total energy intake at an individual level, and consumption of sugar-sweetened beverages should be minimized. These recommendations are much more restrictive than those given by the US, whose guidelines say added sugars should not be more than 25% of energy intake [32] and the European Food Safety Authority, who concluded there was insufficient evidence to set an upper limit for added sugar intakes [33].

### 2.2. Dietary Fructose

The role of dietary fructose in health has been questioned in part because its consumption in the form of high-fructose corn syrup (HFCS; also referred to as glucose-fructose syrup or isoglucose) has increased dramatically in the United States since its introduction in 1967, and this increase has occurred roughly in parallel with the increase in obesity and metabolic disease [34]. In addition to HFCS, fructose is found in the diet in fruit and honey and, along with glucose, as a component of the disaccharide sucrose. However, although mean daily intakes of fructose (49 g/day across all age and gender groups) increased a small amount over the last 40 years in the United States, they alone do not explain the dramatic increase in daily energy and carbohydrate intakes over the same period [35]. The most recent data shows that between 1999 and 2008 the consumption of added sugars in the United States actually decreased from a mean of 100 g/day to 77 g/day [36]. This was linked primarily to a reduced consumption of sugar-sweetened beverages; however overall intakes, contributing to 15% of energy intake, are still much higher than dietary recommendations. 

In the United Kingdom (UK) and Europe, although mean total sugar intakes are high at approximately 100 g/day (20%–25% of energy intake) in adults and children over 4 years, over 50% of the sugar consumed is sucrose, and mean intakes of glucose and fructose (15–18 g/day) are much lower than those in the United States [37,38]. The European Union (EU) is the largest producer of beet sugar and current agricultural policies limit the production of HFCS, so sugar-sweetened beverages in the EU are sweetened primarily with sucrose from beet sugar. 

A recent raft of systematic reviews with meta-analyses have examined the effect of fructose intakes on cardio-metabolic risk factors, including blood lipids [39,40,41], blood pressure [40,42] and body weight [43]. Conclusions are equivocal and influenced largely by fructose dose and hypercaloric energy intakes. For instance, Zhang and colleagues show no effect on total cholesterol and LDL cholesterol when fructose intakes were less than 100 g/day [39]. Similarly, David Wang *et al.* observed no effect of isocaloric fructose substitution at median intakes less than 20% of energy on post prandial triglycerides (TG), but observed a postprandial TG-raising effect in hypercaloric trials where fructose provided 25% excess energy above the background diet [41]. Likewise fructose had no effect on body weight in isocaloric trials, but was associated with weight gain in hypercaloric trials that used more than 100 g/day fructose [43]. All authors commented on the heterogeneity and weak quality of trials included, with small sample sizes and short duration the rule [39,40,41,42,43]. Of note, the SACN carbohydrate working group noted the paucity of trials on fructose that met their inclusion criteria (randomization and duration longer than six weeks) and concluded “there is a lack of evidence to draw conclusions on the impact of sugars intake on the majority of cardio-metabolic outcomes in adults, including body weight” [29]. Here we focus on studies that have implicated dietary sugar, in particular sugar-sweetened beverages and fructose on liver fat and NAFLD pathogenesis.

## 3. Molecular Evidence for a Differential Role for Fructose in NAFLD Pathogenesis 

### 3.1. Transcriptional Regulation of Lipogenic Enzyme Expression

Classic nutrition feeding studies in rats from Naismith [44], Yudkin [45] and others [46] established a differential effect of dietary sugars on the activity of lipogenic enzymes, liver fat and fasting serum TG levels. Using diets where 65%–75% of energy was derived from glucose, fructose, sucrose or starch, these studies showed that fructose alone increased the activity of lipogenic enzymes, including fatty acid synthase, and increased serum and hepatic TG levels. Landmark tracer studies on perfused rat livers demonstrated that fructose, but not glucose, increased the esterification of fatty acids and increased very low density lipoprotein (VLDL)-TG secretion from the liver [47,48]. Many decades of work, as reviewed by Mayes [49], established that observed fructose-induced perturbations of hepatic carbohydrate and lipid metabolism were due to it by-passing the phosphofructokinase rate limiting step in glycolysis. In contrast to glucose, fructose is first rapidly phosphorylated by fructokinase to fructose 1 phosphate, then split into trioses by the activity of aldolase prior to converging with glucose metabolism.

With the advent of the molecular biology era came a focus on the transcriptional response to dietary sugars and much progress has been made in understanding the regulation of lipogenic gene expression. Early work established the induction of the mRNA for pyruvate kinase by fructose [50], and indeed it was analysis of the pyruvate kinase promoter that led to the seminal identification of the carbohydrate response element [51]. The identification of the sterol regulatory element-binding protein 1 (SREBP-1) [52] and the carbohydrate-responsive element-binding protein (ChREBP) [53] has precipitated decades of work characterizing their roles as the major transcriptional regulators of lipogenesis in the liver. Regulation of the activity of the three SREBP isoforms (SREBP-1a, SREBP-1c and SREBP2) and ChREBP is intricate, involving both transcriptional and post-transcriptional mechanisms. However, it is now established that the induction of hepatic glycolytic and lipogenic gene transcription by insulin and glucose is largely mediated by SREBP-1c, and ChREBP respectively, but involves crosstalk with many nutrient-sensitive nuclear receptors [54,55,56,57,58].

Fructose also induces both SREBP-1c and ChREBP activities; the induction of SREBP-1c by fructose has been shown through animal knock-out experiments to be intriguingly dependent on the enzyme stearoyl-CoA desaturase (SCD) and its production of endogenous oleate [59]. More recently, work using antisense oligonucleotides (ASO) has demonstrated that induction of SREBP-1c by fructose is also dependent on the peroxisome proliferator-activated receptor gamma coactivator-1 beta (PGC-1beta), a transcriptional coactivator for SREBP-1 [60]. PGC-1β ASO treatment reduced the expression of SREBP-1c and downstream lipogenic genes and improved the metabolic profile of rats fed a 60% fructose diet for four weeks. Nagai *et al.* showed that the PGC-1β knockdown decreased the occupancy of the SREBP-1c promoter by SREBP1 and the liver X receptor (LXR). The mechanism for how fructose induces ChREBP activity is less clear than that of glucose, but ChREBP knockout mice are markedly intolerant to fructose, dying within a few days of being fed a high fructose diet [61]. Interestingly, while ChREBP knockdown by ASO treatment reduced lipogenic gene expression and plasma TG levels in high-fructose fed rats it did not alter hepatic lipid (TG or DAG) content [62]. The knockdown of ChREBP appeared to reduce DNL by 30%, assessed by deuterium incorporated into palmitate TG, but this was not significant (*p* = 0.1). Importantly, ChREBP, SREBP-1c and LXR have all been shown to be expressed at higher levels in NAFLD patients [63,64,65,66].

In addition to ChREBP and SREBP-1c, relatively recent research has identified the X-box binding protein 1 (XBP1) as a novel transcription factor also regulating hepatic lipogenesis [67]. Conditional knockout of XBP1 in mouse liver reduced DNL, TG secretion and plasma TG levels without causing steatosis. XBP1 was strongly induced in mice fed a 60% fructose diet for 7 days and was shown by chromatin immunoprecipitation assays to directly activate a subset of key lipogenic genes including SCD, diacylglycerol acetyltransferase 2 and acetyl CoA carboxylase 2. As SCD is also induced by SREBP-1c [59], the interplay between nutrient signaling, the transcription factors regulating hepatic lipogenesis, their coactivators, corepressors and target genes is clearly complex. Novel systems biology tools permit the analyses of large-scale gene regulatory and metabolic networks and offer the promise of yielding a mechanistic understanding of their disruption in disease and the development of network-based drugs with fewer adverse effects [68]. Although these tools have been underused in NAFLD research to date [69], it is hoped that future application of systems approaches will further our understanding of the contribution of dietary sugars to disease progression.

### 3.2. Uric Acid

Uric acid is the end product of purine catabolism typically excreted in the urine. An imbalance in the production and excretion of uric acid leads to hyperuricemia and, in some individuals, gout or urolithiasis. Hyperuricemia has been associated with a variety of diseases including chronic kidney disease, obesity, hypertension, and cardiovascular events; however, as recently concluded by Gustafsson [70], the available data preclude a causal relationship. The hyperuricemic effect of fructose was first observed in children with and without hereditary fructose intolerance given fructose intravenously at 0.5 g/kg bodyweight [71]. Subsequent infusion studies in monkeys and human adult males confirmed that high doses of fructose but not glucose, galactose or mannose caused a rapid increase in plasma and urine uric acid levels [72,73]. This is a result of alterations in purine metabolism as a result of the rapid phosphorylation of fructose by fructokinase to fructose 1 phosphate, which leads to a sharp decrease in hepatic ATP levels [49]. In contrast to fructose infusion, the relationship between dietary fructose and serum uric acid levels is less clear. At a population level, US males, but not females, with the highest intakes of added sugars and sugar-sweetened beverages have higher plasma uric acid levels [74]. However in a separate cross-sectional study, although consumption of sugar-sweetened beverages was positively associated with plasma urate, in multivariate analysis fructose intake was not [75]. In addition, a meta-analysis of fructose feeding trials and serum uric acid concluded that energy is confounding factor; with isocaloric fructose having no effect but hypercaloric supplementation of extremely large fructose doses (>200 g/day) increasing serum uric acid levels [76]. The results of Zgaga and colleagues [75] show that plasma urate levels also relate to the amounts of dairy, calcium and meat in the diet; highlighting that sugar-sweetened beverages or fructose may be proxy markers of a poorer diet overall. 

Case-control and cross-sectional studies have observed elevated serum uric acid levels to be independently associated with NAFLD in both children and adults [77,78,79,80,81,82]; while one prospective study in a large (*n* = 6890) Chinese population has shown a linear relationship between baseline serum uric acid levels and incidence of NAFLD after three years [83]. In an observational study that linked higher dietary fructose levels to histological severity of NAFLD, uric acid levels were associated with fructose consumption [84]. However, cause and effect between fructose intake, serum uric acid levels and liver fat or NAFLD severity is not entirely clear cut. Vos and colleagues reported that in children with NAFLD, while uric acid was increased in patients with definite NASH, there was no difference in sugar sweetened beverage consumption between the subgroups of patients [85]. While, in a study by Johnston *et al.* [86], a high fructose (25% of energy) intervention elevated serum uric acid levels and a high glucose intervention lowered serum uric acid levels, there was no change in liver TG levels when this was done isocalorically. When the intervention was given in a hypercaloric fashion, liver TG levels increased in both groups.

Mechanistically, the equivalent elevation of TG by glucose observed by Johnston might be explained in the context of a recent study in animals suggesting endogenous production of fructose from glucose drives fructose-induced fatty liver [87]. Building on previous work [88], Lanaspa and colleagues show that mice lacking either the fructokinase or aldose reductase genes are protected from developing hepatic steatosis [87]. Aldose reductase metabolizes glucose to sorbitol which is then oxidized to fructose in the “polyol pathway”. These data are interesting in light of other work from the same group showing that uric acid activated fructokinase gene expression in a ChREBP dependent fashion [21] and uric acid associated mitochondrial stress led to increased DNL and TG accumulation in hepatocytes *in vitro* [20]. Although preclinical, these mechanistic studies give weight to the argument that uric acid has an independent and deleterious effect on hepatic lipid metabolism and raise the question of whether allopurinol, or other uric acid lowering agents, may be useful in the treatment of NAFLD.

## 4. Dietary Sugar and NAFLD

### 4.1. Observational Data Associating Sugar Intake and NAFLD

In addition to mechanistic studies, several observational studies have established a link between fructose consumption and the presence and progression of NAFLD. Case-control studies using NAFLD patients and BMI-, age- and sex-matched controls have reported more than double the intake of fructose in NAFLD patients relative to the control group, as determined by self-reported soft drink consumption [89,90,91]. In addition, of several biochemical and dietary variables, fructose consumption was found to be the best predictor of the presence of NAFLD in some cohorts [89,90]. Likewise, Zelber-Sagi *et al.* found significantly higher carbohydrate consumption from soft drinks in ultrasound-diagnosed NAFLD patients compared to controls (23 *vs.* 12 g/day) [92], while in patients diagnosed with or without NAFLD during liver resections or biopsies, the intakes of those identified with NAFLD pathology had significantly higher fructose intakes than those without (52 *vs.* 42 g/day) [93]. However, in both studies NAFLD patients tended to have higher energy intake overall, although these differences were not significant. In addition, neither study matched cases with controls, resulting in a higher average BMI and more males in the NAFLD groups [92,93].

While the severity of fatty liver assessed by ultrasound was associated with higher soft drink consumption in the Israeli cohorts of Assy *et al.* and Abid *et al.* [89,90], Abdelmalek and colleagues were the first to examine the association between fructose consumption and NAFLD histological grading [84]. Using ordinal logistic regression models they showed that dietary consumption of seven or more servings of fructose per week was associated with significantly higher histological grades of fibrosis (cumulative odds ratio and 95% confidence interval: 2.6 [1.4, 5.0]), and lower histological grades of steatosis (0.4 [0.2, 0.9]). Notably, patients reporting the highest fructose intake had significantly elevated serum uric acid levels and consumed many more total calories and calories from all macronutrients than those who reported consuming either no fructose or less than seven servings per week.

However, not all observational studies have found a positive association between fructose intake and NAFLD. A cross-sectional study in American children with NAFLD found no association between sucrose-sweetened beverage intake and histological steatosis, NASH or ballooning severity [85]; and in a large Finnish adult population fructose consumption was inversely associated with NAFLD risk defined by the algorithm tests “Fatty Liver Index” and the “NAFLD fat score” [94]. However, all data should be interpreted in view of the limitations of observational studies [95]. In addition, most studies use self-reported beverage intake as a proxy for fructose consumption, which may result in misreporting, miss other key sources of fructose in the diet and, as already mentioned, may potentially act as a marker for an unhealthy diet and lifestyle.

### 4.2. Short-Term Sugar Interventions (≤7 days) and Liver Fat 

**Figure 1:**
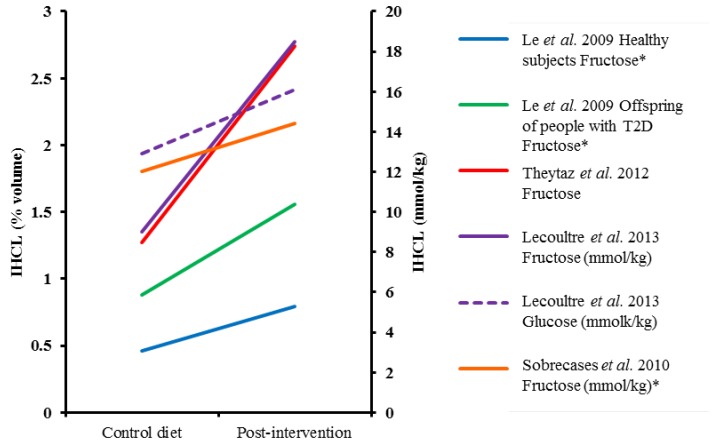
The effect of hypercaloric monosaccharide feeding on IHCL over seven days or less. * Values estimated from figures, T2D; type 2 diabetes, IHCL; intrahepatocellular lipid.

In order to determine causality between the associations found between fructose consumption and NAFLD, several dietary interventions have been undertaken. Over a short time period (6–7 days), fructose feeding of 3–3.5 g/kg/day (approximately 30%–35% of total energy intake) in solution in addition to a control diet has consistently been shown to increase intrahepatocellular lipid (IHCL) compared to the control diet alone, with changes ranging from 16% to 115% [15,96,97,98,99] (Figure 1, Table 1). However, most studies have administered fructose without an isocaloric comparator; therefore, it is unclear whether effects result from fructose per se or simply excessive energy consumption. In order to address these issues, Ngo Sock *et al.* performed a crossover study with a control diet alone, or in combination with 3.5 g/kg/day fructose or glucose [96]. When values at the end of each condition were compared, fructose resulted in a significant increase in IHCL (52%) compared to the control diet, but variation in response to glucose meant that the 58% increase noted after seven days was not significant. In contrast, a recent study showed that feeding 3 g/kg/d of either fructose or glucose over 6–7 days significantly increased IHCL compared to a control diet, and when expressed relative to control values and directly compared, the increase in liver fat after fructose ingestion was significantly higher than after glucose ingestion (113% *vs.* 59%) [99]. Therefore, although the available literature suggests ingesting large quantities of fructose hypercalorically over short time periods does enhance lipid storage, whether this is as a result of fructose metabolism itself or simply increased substrate supply is less clear.

**Table 1:** Summary of study characteristics of short-term (≤7 days) monosaccharide feeding trials and IHCL outcome.

Reference	Participant Characteristics	Study Design	Duration of Intervention	Intervention Dose	Dose as % Energy Requirement	Comparator	Assessment, Units	% Change IHCL Post-Intervention	Significance
Le *et al.*, 2009 [15]	HM (*n* = 8) & male o/s T2D (*n* = 16), mean age 24 year (HM) and 25 year (o/s T2D), mean weight 71 kg (HM) and 75 kg (o/s T2D)	Randomized cross-over	7 days (4–5 week washout)	3.5 g/kg/day fructose	+35%	Control diet	^1^H-MRS, volume %	71% (HM) and 78% (o/s T2D) increase ^§,†^	Absolute change *p* < 0.05 within groups
Lecoultre *et al.*, 2013 [99]	Males (17 in fructose and 11 in glucose group), 23 year, 72 kg, mean BMI 22 kg/m^2^	Randomized cross-over	6–7 days (≥4 week washout)	3 g/kg/day fructose or glucose	+~31% *	Control diet	^1^H-MRS, mmol/kg	113% (F) and 59% (G) increase	Absolute change *p* < 0.05 within groups, % change *p* < 0.05 between groups
Ngo Sock *et al.*, 2010 [96]	Males (11), 25 year, 72 kg	Randomized cross-over	7 days (2–3 weeks washout)	3.5 g/kg/day fructose or glucose	+~36% *	Control diet	^1^H-MRS, mmol/kg	52% (F) and 58% (G) increase	Fructose *p* < 0.05 for % change *vs.* CD
Sobrecases *et al.*, 2010 [98]	Males (12), 24 year, 23 kg/m^2^	Cross-over	7 days	3.5 g/kg fat-free mass/d	+35%	Control diet	^1^H-MRS, mmol/kg	16% increase	*p* < 0.05 *v*s. CD (% change)
Theytaz *et al.*, 2012 [97]	Males (9), 23 year, 23 kg/m^2^	Randomized cross-over	6 days (4–10 weeks washout)	3 g/kg/day fructose	+~30% *	Control diet	^1^H-MRS, volume %	116% increase ^†^	*p* < 0.05 *vs.* CD (absolute change)

* Values estimated from SACN Dietary Reference Values for Energy [100] based on gender, age and weight of participants. ^§^ Values estimated from figures. ^†^ %changes calculated from absolute values. IHCL: intra-hepatocellular lipid; HM: healthy males; o/s T2D: offspring of people with type 2 diabetes; CD: control diet; ^1^H-MRS: proton-magnetic resonance spectroscopy; F: fructose; G: glucose.

### 4.3. Long-Term Sugar Feeding and Liver Fat

**Figure 2:**
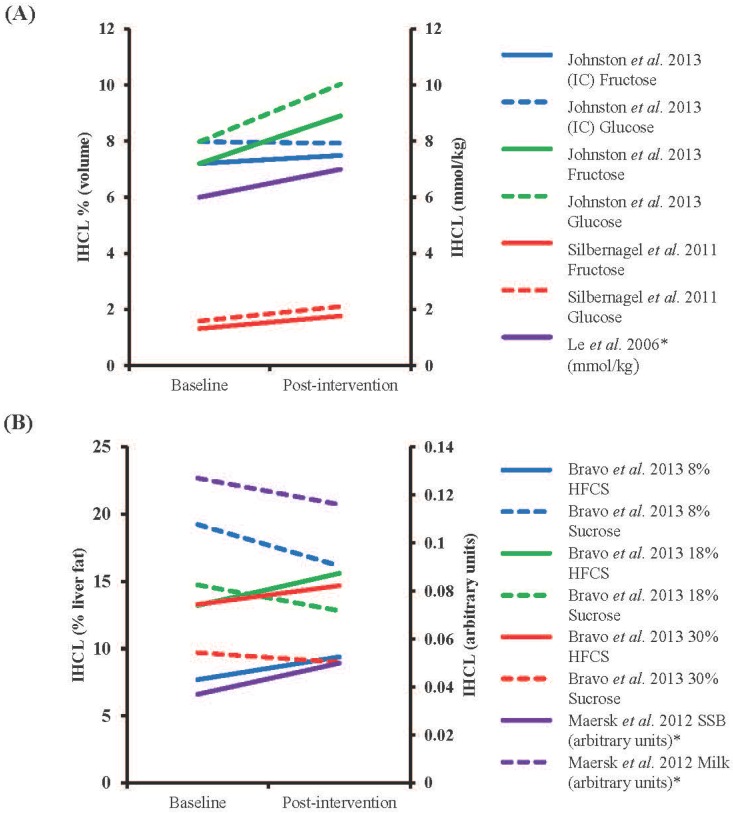
The effect of isocaloric (IC) and hypercaloric monosaccharide feeding on IHCL over more than seven days. (**A**) Changes in interventions using fructose and/or glucose. Interventions were hypercaloric unless otherwise stated (**B**) Changes in interventions using alternative fructose-containing solutions and comparators (Bravo *et al.* [104]; isocaloric high-fructose corn syrup (HFCS) *vs.* sucrose, Maersk *et al.* [103]; hypercaloric sucrose-sweetened beverage (SSB) *vs.* milk). * Values estimated from figures, IHCL; intrahepatocellular lipid.

In contrast to short-term feeding studies, results of long-term interventions are more mixed (Table 2, Figure 2). When provided in addition to baseline energy intake, evidence of a deleterious effect of fructose is inconclusive; an additional 18%–25% of energy consumed as pure fructose by healthy subjects over four weeks in the studies of Le *et al.* [101] and Silbernagel *et al.* [102] failed to result in an increase in IHCL compared to baseline. In contrast, in centrally obese men, when fructose or glucose was provided as an additional 25% energy intake for two weeks, significant increases in IHCL compared to baseline were observed; however, there were no differences between the groups consuming either glucose or fructose (IHCL increased by 26% and 24%, respectively) [86]. In a separate study, a significant increase in liver fat was observed in overweight adults who consumed 106 g/day sucrose in the form of a sweetened beverage for six months, but not in matched subjects who consumed an equivalent amount of energy as milk [103]. Although intriguing, given the vastly different macro- and micronutrient composition of the beverages, these data are somewhat difficult to interpret. Other isocaloric trials have failed to demonstrate an adverse effect of fructose on IHCL. For example, in healthy adults computed tomography scans showed no difference in liver fat after 10 weeks of 55% HFCS consumption accounting for 8, 18 or 30% of energy intake compared to sucrose or baseline values [104], while substitution of 25% energy in a controlled diet with glucose or fructose resulted in no differences in IHCL [86]. Overall, neither hypercaloric nor isocaloric interventions over more than seven days demonstrate a definitive effect of fructose on increasing liver fat compared to baseline, or an isocaloric comparator.

### 4.4. Interventions Aimed at Reducing Fructose Intakes and Liver Fat

In addition to studying the effect of the addition of fructose on IHCL profiles, another approach has been to reduce fructose intake and examine hepatic lipid content. A decrease in total fructose consumption by 50% for six months resulted in a reduction of liver fat in adults with NAFLD [105]. However, it is unclear whether these results were a direct result of fructose reduction or an overall change in dietary composition and energy restriction. In an alternative design, Jin *et al.* recruited Hispanic-American adolescents who were already consuming three sucrose-sweetened beverages per day and replaced them with either glucose or fructose sweetened beverages for four weeks. They found no difference between IHCL levels in those continuing with fructose-containing beverages and those consuming glucose only, suggesting that replacing fructose with glucose did not improve hepatic lipid storage over this time period [106]. Again, these data do not provide a conclusive role for fructose in NAFLD independent from glucose or other forms of sugar. However, better controlled trials of this nature would allow a meaningful and physiologically relevant intervention in people with NAFLD and may help to further elucidate any association between fructose consumption and IHCL.

Taken together, intervention results do not provide sufficient evidence that fructose acts a significant lipogenic precursor, especially when compared directly with glucose, a conclusion supported by two recent meta-analyses, mentioned previously [22,23]. Some of this may be due to the heterogeneous nature of these interventions; in long term studies duration ranges from two weeks to six months, with fructose delivered alone, or as part of HFCS or sucrose, at either hyper or isocaloric levels with the habitual diet (Table 2). This is in contrast to a largely homogenous group of participants, namely young, healthy males [22,23]. In addition, most studies supplied the intervention solution with a meal; however, a recent study over 6 weeks suggests that sucrose-sweetened beverages are more lipogenic when consumed in-between, rather than with meals [107]. Therefore, it seems there is more research required to address the impact on timing of beverages, as well as some of the issues of: insufficient study duration and participant numbers, lack of isocaloric comparators, hypercaloric feeding and the limited participant demographic, in order to provide a conclusive role for fructose at levels relevant to population intake in hepatic fat accumulation.

**Table 2:** Summary of study characteristics of long-term monosaccharide feeding trials and IHCL outcome.

Reference	Participant characteristics	Study design	Duration of Intervention	Intervention Dose	Dose as % Energy Requirements	Comparator	Assessment, Units	% Change IHCL Post-Intervention	Significance
Bravo *et al.*, 2013 [104]	Males and females (*n* = 64 in total), mean age 42.2 year, mean BMI 23–35 kg/m^2^	Randomized, partially blinded, parallel intervention	10 weeks	8%, 18% or 30% of energy intake as HFCS or sucrose	8%, 18% or 30%	Baseline	CT, % liver fat content	22, 18 and 11% increase (HFCS) and 16, 13 and 7% decrease (S) for 8, 18 and 30% supplementation ^†^	NSD in absolute changes in any group
Johnston *et al.*, 2013 [86]	Centrally obese males (15 in fructose and 17 in glucose group), 35 year (F) and 33 year (G), 30 kg/m^2^ (F) and 28.9 kg/m^2^ (G)	Randomized parallel intervention	2 weeks	25% of energy intake as glucose or fructose (IC) or same weight in addition to control diet (HC)	25% (IC) or +25% (HC)	Baseline	^1^H-MRS, volume %	After IC condition 4% increase (F) and 1% decrease (G), after HC condition 24% (F) and 26% (G) increase ^†^	NSD after IC diet, absolute values of fructose and glucose *p* < 0.05 *vs.* baseline
Le *et al.*, 2006 [101]	Males (7), 24.7 year, mean weight 69.3 kg	Parallel intervention	4 weeks	1.5 g/kg/day	+18%	Baseline	^1^H-MRS, mmol/kg	17% increase ^§,†^	NSD in absolute changes
Maersk *et al.*, 2012 [103]	Males and females (10 in SSB group and 12 in milk group), 39 year (SSB) and 38 year (milk), 31.3 kg/m^2^ (SSB) and 31.9 kg/m^2^ (milk)	Randomized parallel intervention	6 months	106 g/day SSB or equivalent energy as milk	+~16% (males) and +~20% (females) *	Baseline	^1^H-MRS, arbitrary units	36% increase after SSB, 9% decrease after milk ^§^	*p* < 0.05 for % change between SSB and milk
Silbernagel *et al.*, 2011 [102]	Males and females (10 in fructose and 10 in glucose groups), 30.5 year, 25.9 kg/m^2^	Randomized, single-blinded parallel intervention	4 weeks	150 g/day fructose or glucose	+~25%	Baseline	^1^H-MRS, volume %	34% (F) and 33% (G) increase ^†^	NSD in absolute changes

* Values estimated from SACN Dietary Reference Values for Energy [100] based on the gender and mean age and weight of participants. ^§^ Values estimated from figures. ^†^ %changes calculated from absolute values. IHCL: intra-hepatocellular lipid; HFCS: High-fructose corn syrup; IC: isocaloric; CT: computed tomography; S: sucrose; NSD: no significant differences; F: fructose; G: glucose; HC: hypercaloric; ^1^H-MRS: proton-magnetic resonance spectroscopy, SSB: sucrose-sweetened beverage.

## 5. Measurement of Hepatic *De novo* Lipogenesis 

Fat in the liver may arise from many sources, including dietary fat as discussed by Green and Hodson in a separate review paper in this edition [108]. Several studies have investigated the extent to which carbohydrate-induced DNL may be causal in the development of NAFLD and we will focus on those here. *De novo* lipogenesis is the term used for the synthesis of fatty acids from non-lipid precursors such as fructose, glucose or amino acids. To estimate DNL *in vivo*, several different stable isotope approaches may be used and it is worth taking the time to consider the relative merits of each method in order to interpret data from the literature. If liver samples cannot be accessed, then VLDL-TG is used as a proxy marker of the hepatic TG pool. VLDL-TG can be specifically isolated using techniques such as immunoaffinity chromatography [109]. Otherwise, a TG-rich lipoprotein fraction (usually “Sf 20-400”) is taken but this also contains chylomicron remnants. Studies that do not separate intestinal from hepatic lipoproteins (e.g., Chong *et al.* [110], Parks *et al.* [13], Donnelly *et al.* [111]) therefore include measurements of intestinal DNL. It should be noted that the release of non-esterified fatty acids from adipose tissue (formed by DNL) could indirectly contribute to the measurement of DNL in liver and VLDL-TG. As previously discussed by others [110], this is thought to be quantitatively minor but has not been directly measured.

It is important to note the main product of DNL in humans is palmitate and most researchers only monitor the appearance of the stable isotope label in palmitate-TG. Although the result is usually expressed as “%DNL”, it does not equate to the total TG pool that has arisen by DNL. Therefore, the term “%DNL” although commonly used, may be somewhat misleading if not understood correctly. Recently, this point was illustrated by Lambert *et al.* [112] who measured fatty acid synthesis in VLDL-TG to estimate hepatic DNL in people with type 1 diabetes and controls. They comprehensively measured DNL in the different VLDL-TG fatty acids and reported that the mean %DNL in palmitate in the control group was 14% whereas it was 2% for oleic acid. This agrees with earlier work that found that the proportion of newly synthesized stearate (18:0) was less than half that measured for palmitate [113]. Taking into account fatty acid concentration, Wilke *et al.* [114] found that palmitate, followed by 18:1 fatty acids, and then myristate were quantitatively the major fatty acids formed by *de novo* lipogenesis. Thus, %DNL is an important measure of *de novo* lipogenesis, which usually refers specifically to palmitate, the main product of DNL.

### 5.1. Deuterated Water

Since water is used in the synthesis of fatty acids, deuterated water (^2^H_2_O) can be used to calculate the fractional synthesis of fatty acids. Deuterated water is either given orally (*in vivo*) or in the cell media (*in vitro*) and the appearance of ^2^H in the fatty acid acyl chain is determined, allowing for the relative proportion of hydrogens donated by NADPH or H_2_O.

### 5.2. [^13^C]acetate

[^13^C]acetate can also be used to calculate DNL and the method is based on equilibration in the precursor pool of acetyl CoA, the building block of fatty acid synthesis. Labelling of the precursor pool is a clear advantage of the method, but the protocol requires a fairly long intravenous infusion time. A seminal piece of work in this area was that of Hellerstein *et al.* who used a thorough mathematical approach (mass isotopomer distribution analysis, MIDA) to show that DNL was quantitatively very low after an overnight fast (less than 1%) in healthy humans [113].

### 5.3. [^13^C]sugars

In order to assess the contribution of dietary sugars to DNL, sugars labelled with ^13^C have been traced into TG-fatty acids in a limited number of studies. Whereas the approaches above using deuterated water and [^13^C]acetate measure %DNL from all precursor sources, it should be noted that tracing the incorporation of dietary sugars into TG will not include the upregulation of DNL from other sources (*i.e.*, the precursor pool is not labelled). Therefore the use of [^13^C] sugars does not measure % DNL but rather gives an assessment of any changes/differences in the DNL pathway. 

### 5.4. Fatty Acid Profile

The fatty acid composition of liver-TG or VLDL-TG has been used to infer the extent of DNL in human studies without the need for isotope labelling. Hudgins *et al.* [115] used an ingenious technique whereby they matched the fatty acid composition of the diet to an individuals’ adipose tissue composition so that the essential fatty acid 18:2 *n*-6 could be used as a non-isotopic marker of non-DNL fatty acids in VLDL-TG. Thus, fatty acids formed de novo were calculated from the dilution of 18:2 *n*-6 in VLDL-TG relative to the composition in the diet. A limitation is that the method requires an adaptation period and controlled feeding, therefore it is not the most appropriate choice for cross-sectional or short-term feeding studies. A point to note, regarding the use of hepatic fatty acid composition in relation to liver fat accumulation, is that it is essential to use a single lipid pool (*i.e.*, TG). It has been elegantly shown by Peter *et al.* [116] that as liver accumulates TG, the ratio of TG to phospholipid increases and the relative proportions of different fatty acids in the total lipid pool change such that the ratio of 18:1 *n*-9/18:0 increases simply because the fatty acid composition of the two lipid pools are different. Therefore the results of studies that have measured fatty acid composition in liver samples in relation to NAFLD or liver fat must be interpreted with caution if specific lipid pools (e.g., TG) have not been sampled [117,118].

Hepatic lipids comprise a complex range of species [119] and TG is the most abundant which accumulates further in simple steatosis. However, liver fat accumulation is characterized by the accumulation of both TG and cholesterol, with TG and cholesteryl ester allowing storage for excess fatty acid moieties [120]. A high fructose ad libitum diet leads to a greater increase in hepatic TG than cholesterol in a mouse model [121]. However, in humans this has not been well studied.

## 6. The Contribution of DNL to Hepatic Lipid Accumulation

### 6.1. Metabolic Fate of Fructose

**Figure 3:**
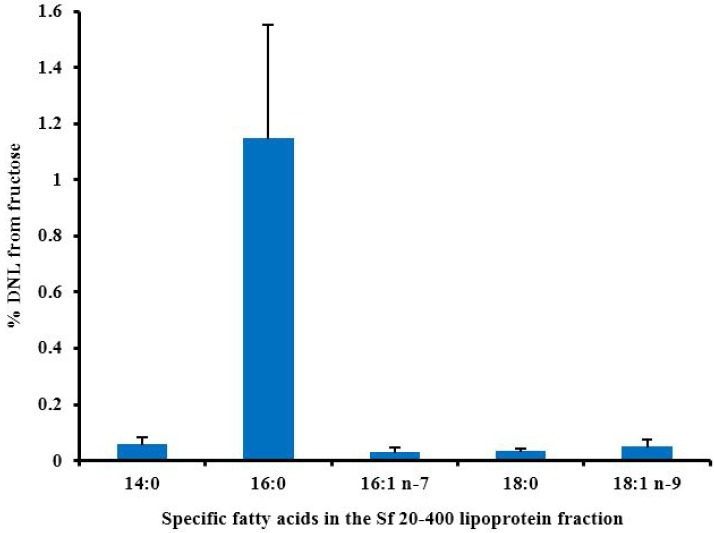
The % of individual fatty acids (±SEM) that have arisen by DNL 4 h after healthy subjects consumed 0.75 g fructose/kg body weight (9% of energy requirement) as part of a mixed liquid meal. Calculated from data collected for Chong *et al.* [110].

Changes in hepatic DNL in response to fructose ingestion have been measured in a few human studies. Chong *et al.* [110] gave [^13^C]glucose or fructose as 0.75 g/kg body weight to healthy participants. The sugars were traced into TG-fatty acids and TG-glycerol, and although significantly more TG-fatty acids were derived from fructose than glucose, in the short term only a very small proportion of TG-fatty acids were derived from fructose (<0.5%), compared with 38% TG-glycerol formed *de novo*. Four hours after ingestion, newly synthesized fatty acids from fructose made up less than one percent of circulating VLDL-TG. For those individuals who synthesized detectable amounts of fatty acids, palmitate was the major end point of fatty acid synthesis (Figure 3), in agreement with studies mentioned above. However from the point of view of the metabolic fate of fructose, the results showed that only 0.05% and 0.15% of fructose were converted to TG-fatty acids and TG-glycerol, respectively. Values after glucose ingestion were close to 0%. This work, and other human studies using isotopically labelled tracers have been comprehensively reviewed by Sun and Empie [122]. They concluded that the immediate metabolic fate of ingested fructose was not into plasma TG (<1%) but towards oxidation (45%) and conversion to glucose (41%). Conversion to lactate is also quantitatively important.

### 6.2. The Contribution of DNL to Liver Fat Accumulation

It is difficult to directly assess the impact of DNL on liver fat accumulation because of the inaccessibility of the liver in humans, but Donnelly *et al.* [111] were able to directly assess DNL in obese and morbidly obese individuals undergoing a scheduled liver biopsy. In these individuals, over one quarter of palmitate in liver TG was accounted for by DNL, with a similar pattern in lipoprotein TG, showing that VLDL-TG is a good proxy marker for liver TG. The high level of DNL compared with other studies suggests that DNL may be upregulated in individuals who have been in a state of caloric excess and could contribute to liver fat accumulation in NAFLD. An alternative explanation is a methodological difference between this and other studies; in the Donnelly paper there was a long tracer infusion time, specifically 4 days of [^13^C] acetate infusion. The same group have since compared % DNL and rate of DNL fatty acid synthesis in individuals with low and high liver fat and found that mean values were significantly higher in the high liver fat group (10% *vs.* 23% and 2.6 *vs.* 1.5 µmol/min respectively) during a 12 h period after an evening meal [123] in accord with an earlier, smaller postabsorptive study [124] as discussed in [123].

### 6.3. Hepatic DNL in Response to Fructose

In this section we report studies that have measured %DNL in VLDL-TG as a proxy for liver TG. In an acute setting, healthy individuals consumed 85 g sugar added to a meal. Using [^13^C]acetate to measure DNL, it was shown that DNL was higher when subjects ingested a meal containing 50% glucose and 50% fructose (16%) compared with 8% when the sugar was only glucose [13]. Moreover, after a subsequent meal, there was an increase in plasma VLDL-TG concentrations. Rather than being due to increased DNL per se, it was suggested that the stimulation of DNL created “a metabolic milieu that enhances subsequent esterification of fatty acids flowing to the liver to elevate TG synthesis postprandially”. In another arm of the study, 25% glucose and 75% fructose was given and there was no difference in %DNL compared with the 50:50 arm, suggesting no dose effect, although it may be an issue of insufficient power (*n* = 6 subjects). Stanhope *et al.* [14] measured DNL in subjects who had consumed fructose or glucose sweetened beverages, providing 25% energy, for 10 weeks with significant increases in body weight. Surprisingly, they found a significant increase in fasting plasma TG concentrations after the glucose diet, but not after fructose. However, postprandial lipaemia was higher after the fructose diet compared with baseline but this effect was not seen in the glucose arm. There was a parallel increase in %DNL in the fructose arm which increased from 11 to 17%. There was no significant difference in fasting DNL after the two diets. The study design did not allow any quantitation of the contribution of fructose to the enhanced postprandial lipaemia but in accordance with Parks *et al.* [13], it was suggested that increased fatty acid esterification from other sources may play a role. In response to a high fructose diet (3 g fructose per kg per day given as a supplement in drinks) *vs.* a control diet without the drinks, fasting DNL was significantly higher (9% *vs.* 2%) [125].

The percentage term for DNL may be adjusted for the fatty acid pool size, and when combined with flux data, can give a quantitative result such as mg palmitate in VLDL-TG that has arisen from DNL per day [126], but this has rarely been done in fructose feeding studies. Moreover, in order to precisely quantify of the amount of palmitate synthesized in response to a dietary sugar, the amount that remains in the liver would need to be accounted for. Because of the difficulty of accurately assessing this, it remains unresolved. However, the present evidence suggests a greater effect of fructose than glucose on DNL, particularly in the postprandial period, but more studies are required to show the effect in response to habitually consumed foods containing fructose.

## 7. Conclusions

Despite a large body of work the health and metabolic effects of fructose and other dietary sugars are unresolved. Multiple mechanisms have been described for how large doses of fructose are lipogenic and have adverse metabolic effects. The data taken together suggest that although fructose does stimulate DNL to some extent, quantitatively the lipogenic effect of fructose is not mediated exclusively by its provision of excess substrates for DNL. The deleterious metabolic effects of supra-physiological fructose loads are a consequence of altered transcriptional regulatory networks impacting intracellular macronutrient metabolism, including fatty acid oxidation and esterification, along with altering signaling and inflammatory processes. Increased production of uric acid may also contribute to or exacerbate these effects. Pragmatically, in the context of epidemic levels of obesity, reducing dietary sugar consumption is a prudent public health message. However, whether or not dietary sugars, including fructose, at typically consumed population levels, effects hepatic lipogenesis and NAFLD pathogenesis in humans independently of excess energy remains an unresolved question.

## References

[1] Loomba R., Sanyal A.J. (2013). The global NAFLD epidemic. Nat. Rev. Gastroenterol. Hepatol..

[2] Machado M., Marques-Vidal P., Cortez-Pinto H. (2006). Hepatic histology in obese patients undergoing bariatric surgery. J. Hepatol..

[3] Vernon G., Baranova A., Younossi Z.M. (2011). Systematic review: The epidemiology and natural history of non-alcoholic fatty liver disease and non-alcoholic steatohepatitis in adults. Aliment. Pharmacol. Ther..

[4] Moore J.B. (2010). Non-alcoholic fatty liver disease: The hepatic consequence of obesity and the metabolic syndrome. Proc. Nutr. Soc..

[5] Liu C.J. (2012). Prevalence and risk factors for non-alcoholic fatty liver disease in Asian people who are not obese. J. Gastroenterol. Hepatol..

[6] Guerrero R., Vega G.L., Grundy S.M., Browning J.D. (2009). Ethnic differences in hepatic steatosis: An insulin resistance paradox?. Hepatology.

[7] Nascimbeni F., Pais R., Bellentani S., Day C.P., Ratziu V., Loria P., Lonardo A. (2013). From NAFLD in clinical practice to answers from guidelines. J. Hepatol..

[8] Chalasani N., Younossi Z., Lavine J.E., Diehl A.M., Brunt E.M., Cusi K., Charlton M., Sanyal A.J. (2012). The diagnosis and management of non-alcoholic fatty liver disease: Practice Guideline by the American Association for the Study of Liver Diseases, American College of Gastroenterology, and the American Gastroenterological Association. Hepatology.

[9] Neuschwander-Tetri B.A. (2013). Carbohydrate intake and nonalcoholic fatty liver disease. Curr. Opin. Clin. Nutr. Metab. Care.

[10] Bray G.A., Popkin B.M. (2013). Calorie-sweetened beverages and fructose: What have we learned 10 years later. Pediatr. Obes..

[11] Vos M.B., Lavine J.E. (2013). Dietary fructose in nonalcoholic fatty liver disease. Hepatology.

[12] Mancini M., Mattock M., Rabaya E., Chait A., Lewis B. (1973). Studies of the mechanisms of carbohydrate-induced lipaemia in normal man. Atherosclerosis.

[13] Parks E.J., Skokan L.E., Timlin M.T., Dingfelder C.S. (2008). Dietary sugars stimulate fatty acid synthesis in adults. J. Nutr..

[14] Stanhope K.L., Schwarz J.M., Keim N.L., Griffen S.C., Bremer A.A., Graham J.L., Hatcher B., Cox C.L., Dyachenko A., Zhang W. (2009). Consuming fructose-sweetened, not glucose-sweetened, beverages increases visceral adiposity and lipids and decreases insulin sensitivity in overweight/obese humans. J. Clin. Investig..

[15] Le K.A., Ith M., Kreis R., Faeh D., Bortolotti M., Tran C., Boesch C., Tappy L. (2009). Fructose overconsumption causes dyslipidemia and ectopic lipid deposition in healthy subjects with and without a family history of type 2 diabetes. Am. J. Clin. Nutr..

[16] Bergheim I., Weber S., Vos M., Kramer S., Volynets V., Kaserouni S., McClain C.J., Bischoff S.C. (2008). Antibiotics protect against fructose-induced hepatic lipid accumulation in mice: Role of endotoxin. J. Hepatol..

[17] Kawasaki T., Igarashi K., Koeda T., Sugimoto K., Nakagawa K., Hayashi S., Yamaji R., Inui H., Fukusato T., Yamanouchi T. (2009). Rats fed fructose-enriched diets have characteristics of nonalcoholic hepatic steatosis. J. Nutr..

[18] Tappy L., Le K.A. (2010). Metabolic effects of fructose and the worldwide increase in obesity. Physiol. Rev..

[19] Samuel V.T. (2011). Fructose induced lipogenesis: From sugar to fat to insulin resistance. Trends Endocrinol. Metab..

[20] Lanaspa M.A., Sanchez-Lozada L.G., Choi Y.J., Cicerchi C., Kanbay M., Roncal-Jimenez C.A., Ishimoto T., Li N., Marek G., Duranay M. (2012). Uric acid induces hepatic steatosis by generation of mitochondrial oxidative stress: Potential role in fructose-dependent and -independent fatty liver. J. Biol. Chem..

[21] Lanaspa M.A., Sanchez-Lozada L.G., Cicerchi C., Li N., Roncal-Jimenez C.A., Ishimoto T., Le M., Garcia G.E., Thomas J.B., Rivard C.J. (2012). Uric acid stimulates fructokinase and accelerates fructose metabolism in the development of fatty liver. PLoS One.

[22] Chiu S., Sievenpiper J.L., de Souza R.J., Cozma A.I., Mirrahimi A., Carleton A.J., Ha V., di Buono M., Jenkins A.L., Leiter L.A. (2014). Effect of fructose on markers of non-alcoholic fatty liver disease (NAFLD): A systematic review and meta-analysis of controlled feeding trials. Eur. J. Clin. Nutr..

[23] Chung M., Ma J., Patel K., Berger S., Lau J., Lichtenstein A.H. (2014). Fructose, high-fructose corn syrup, sucrose, and nonalcoholic fatty liver disease or indexes of liver health: A systematic review and meta-analysis. Am. J. Clin. Nutr..

[24] Laughlin M.R., Bantle J.P., Havel P.J., Parks E., Klurfeld D.M., Teff K., Maruvada P. (2014). Clinical research strategies for fructose metabolism. Adv. Nutr..

[25] White J.S. (2013). Challenging the fructose hypothesis: New perspectives on fructose consumption and metabolism. Adv. Nutr..

[26] Rippe J.M., Angelopoulos T.J. (2013). Sucrose, high-fructose corn syrup, and fructose, their metabolism and potential health effects: What do we really know?. Adv. Nutr..

[27] Macdonald I.A. (2014). Dietary strategies for the management of cardiovascular risk: Role of dietary carbohydrates. Proc. Nutr. Soc..

[28] WHO (2014). Guideline: Sugars Intake for Adults and Children: Draft Guidelines on Free Sugars Released for Public Consultation.

[29] Scientific Advisory Committee on Nutrition (2014). Draft Carbohydrates and Health Report.

[30] Te Morenga L., Mallard S., Mann J. (2013). Dietary sugars and body weight: Systematic review and meta-analyses of randomised controlled trials and cohort studies. BMJ.

[31] Moynihan P.J., Kelly S.A. (2014). Effect on caries of restricting sugars intake: Systematic review to inform WHO guidelines. J. Dent. Res..

[32] US Department of Agriculture (2010). Dietary Guidelines for Americans, 2010.

[33] European Food Safety Authority (2010). Scientific Opinion on Dietary Reference Values for carbohydrates and dietary fibre. EFSA J..

[34] Van Buul V.J., Tappy L., Brouns F.J. (2014). Misconceptions about fructose-containing sugars and their role in the obesity epidemic. Nutr. Res. Rev..

[35] Marriott B.P., Cole N., Lee E. (2009). National estimates of dietary fructose intake increased from 1977 to 2004 in the United States. J. Nutr..

[36] Welsh J.A., Sharma A.J., Grellinger L., Vos M.B. (2011). Consumption of added sugars is decreasing in the United States. Am. J. Clin. Nutr..

[37] Bates B., Lennox A., Prentice A., Bates C.J., Swan G. (2012). National Diet and Nutrition Survey: Headline Results from Years 1, 2 and 3 (combined) of the Rolling Programme 2008/09–2010/11.

[38] Ahmadi-Abhari S., Luben R.N., Powell N., Bhaniani A., Chowdhury R., Wareham N.J., Forouhi N.G., Khaw K.T. (2014). Dietary intake of carbohydrates and risk of type 2 diabetes: The European Prospective Investigation into Cancer-Norfolk study. Br. J. Nutr..

[39] Zhang Y.H., An T., Zhang R.C., Zhou Q., Huang Y., Zhang J. (2013). Very high fructose intake increases serum LDL-cholesterol and total cholesterol: A meta-analysis of controlled feeding trials. J. Nutr..

[40] Kelishadi R., Mansourian M., Heidari-Beni M. (2014). Association of fructose consumption and components of metabolic syndrome in human studies: A systematic review and meta-analysis. Nutrition.

[41] David Wang D., Sievenpiper J.L., de Souza R.J., Cozma A.I., Chiavaroli L., Ha V., Mirrahimi A., Carleton A.J., di Buono M., Jenkins A.L. (2014). Effect of fructose on postprandial triglycerides: A systematic review and meta-analysis of controlled feeding trials. Atherosclerosis.

[42] Ha V., Sievenpiper J.L., de Souza R.J., Chiavaroli L., Wang D.D., Cozma A.I., Mirrahimi A., Yu M.E., Carleton A.J., Dibuono M. (2012). Effect of fructose on blood pressure: A systematic review and meta-analysis of controlled feeding trials. Hypertension.

[43] Sievenpiper J.L., de Souza R.J., Mirrahimi A., Yu M.E., Carleton A.J., Beyene J., Chiavaroli L., di Buono M., Jenkins A.L., Leiter L.A. (2012). Effect of fructose on body weight in controlled feeding trials: A systematic review and meta-analysis. Ann. Intern. Med..

[44] Naismith D.J. (1971). Differences in the metabolism of dietary carbohydrates studied in the rat. Proc. Nutr. Soc..

[45] Bruckdorfer K.R., Khan I.H., Yudkin J. (1972). Fatty acid synthetase activity in the liver and adipose tissue of rats fed with various carbohydrates. Biochem. J..

[46] Waddell M., Fallon H.J. (1973). The effect of high-carbohydrate diets on liver triglyceride formation in the rat. J. Clin. Investig..

[47] Topping D.L., Mayes P.A. (1972). The immediate effects of insulin and fructose on the metabolism of the perfused liver. Changes in lipoprotein secretion, fatty acid oxidation and esterification, lipogenesis and carbohydrate metabolism. Biochem. J..

[48] Topping D.L., Mayes P.A. (1976). Comparative effects of fructose and glucose on the lipid and carbohydrate metabolism of perfused rat liver. Br. J. Nutr..

[49] Mayes P.A. (1993). Intermediary metabolism of fructose. Am. J. Clin. Nutr..

[50] Noguchi T., Inoue H., Tanaka T. (1982). Regulation of rat liver L-type pyruvate kinase mRNA by insulin and by fructose. Eur. J. Biochem..

[51] Thompson K.S., Towle H.C. (1991). Localization of the carbohydrate response element of the rat L-type pyruvate kinase gene. J. Biol. Chem..

[52] Wang X., Sato R., Brown M.S., Hua X., Goldstein J.L. (1994). SREBP-1, a membrane-bound transcription factor released by sterol-regulated proteolysis. Cell.

[53] Yamashita H., Takenoshita M., Sakurai M., Bruick R.K., Henzel W.J., Shillinglaw W., Arnot D., Uyeda K. (2001). A glucose-responsive transcription factor that regulates carbohydrate metabolism in the liver. Proc. Natl. Acad. Sci. USA.

[54] Shao W., Espenshade P.J. (2012). Expanding roles for SREBP in metabolism. Cell Metab..

[55] Postic C., Dentin R., Denechaud P.D., Girard J. (2007). ChREBP, a transcriptional regulator of glucose and lipid metabolism. Annu. Rev. Nutr..

[56] Poupeau A., Postic C. (2011). Cross-regulation of hepatic glucose metabolism via ChREBP and nuclear receptors. Biochim. Biophys. Acta.

[57] Yoshikawa T., Shimano H., Yahagi N., Ide T., Amemiya-Kudo M., Matsuzaka T., Nakakuki M., Tomita S., Okazaki H., Tamura Y. (2002). Polyunsaturated fatty acids suppress sterol regulatory element-binding protein 1c promoter activity by inhibition of liver X receptor (LXR) binding to LXR response elements. J. Biol. Chem..

[58] Caron S., Huaman Samanez C., Dehondt H., Ploton M., Briand O., Lien F., Dorchies E., Dumont J., Postic C., Cariou B. (2013). Farnesoid X receptor inhibits the transcriptional activity of carbohydrate response element binding protein in human hepatocytes. Mol. Cell. Biol..

[59] Miyazaki M., Dobrzyn A., Man W.C., Chu K., Sampath H., Kim H.J., Ntambi J.M. (2004). Stearoyl-CoA desaturase 1 gene expression is necessary for fructose-mediated induction of lipogenic gene expression by sterol regulatory element-binding protein-1c-dependent and -independent mechanisms. J. Biol. Chem..

[60] Nagai Y., Yonemitsu S., Erion D.M., Iwasaki T., Stark R., Weismann D., Dong J., Zhang D., Jurczak M.J., Loffler M.G. (2009). The role of peroxisome proliferator-activated receptor gamma coactivator-1 beta in the pathogenesis of fructose-induced insulin resistance. Cell Metab..

[61] Iizuka K., Bruick R.K., Liang G., Horton J.D., Uyeda K. (2004). Deficiency of carbohydrate response element-binding protein (ChREBP) reduces lipogenesis as well as glycolysis. Proc. Natl. Acad. Sci. USA.

[62] Erion D.M., Popov V., Hsiao J.J., Vatner D., Mitchell K., Yonemitsu S., Nagai Y., Kahn M., Gillum M.P., Dong J. (2013). The role of the carbohydrate response element-binding protein in male fructose-fed rats. Endocrinology.

[63] Benhamed F., Denechaud P.D., Lemoine M., Robichon C., Moldes M., Bertrand-Michel J., Ratziu V., Serfaty L., Housset C., Capeau J. (2012). The lipogenic transcription factor ChREBP dissociates hepatic steatosis from insulin resistance in mice and humans. J. Clin. Investig..

[64] Kohjima M., Enjoji M., Higuchi N., Kato M., Kotoh K., Yoshimoto T., Fujino T., Yada M., Yada R., Harada N. (2007). Re-evaluation of fatty acid metabolism-related gene expression in nonalcoholic fatty liver disease. Int. J. Mol. Med..

[65] Higuchi N., Kato M., Shundo Y., Tajiri H., Tanaka M., Yamashita N., Kohjima M., Kotoh K., Nakamuta M., Takayanagi R. (2008). Liver X receptor in cooperation with SREBP-1c is a major lipid synthesis regulator in nonalcoholic fatty liver disease. Hepatol. Res..

[66] Ahn S.B., Jang K., Jun D.W., Lee B.H., Shin K.J. (2014). Expression of Liver X Receptor Correlates with Intrahepatic Inflammation and Fibrosis in Patients with Nonalcoholic Fatty Liver Disease. Dig. Dis. Sci..

[67] Lee A.H., Scapa E.F., Cohen D.E., Glimcher L.H. (2008). Regulation of hepatic lipogenesis by the transcription factor XBP1. Science.

[68] Plant N.J. (2014). An introduction to systems toxicology. Toxicol. Res..

[69] Fisher C.P., Kierzek A.M., Plant N.J., Moore J.B. (2014). Systems biology approaches for studying the pathogenesis of non-alcoholic fatty liver disease. World J. Gastroenterol..

[70] Gustafsson D., Unwin R. (2013). The pathophysiology of hyperuricaemia and its possible relationship to cardiovascular disease, morbidity and mortality. BMC Nephrol..

[71] Perheentupa J., Raivio K. (1967). Fructose-induced hyperuricaemia. Lancet.

[72] Simkin P.A. (1972). Hexose infusions in Cebus monkeys: Effects on uric acid metabolism. Metabolism.

[73] Narins R.G., Weisberg J.S., Myers A.R. (1974). Effects of carbohydrates on uric acid metabolism. Metabolism.

[74] Gao X., Qi L., Qiao N., Choi H.K., Curhan G., Tucker K.L., Ascherio A. (2007). Intake of added sugar and sugar-sweetened drink and serum uric acid concentration in US men and women. Hypertension.

[75] Zgaga L., Theodoratou E., Kyle J., Farrington S.M., Agakov F., Tenesa A., Walker M., McNeill G., Wright A.F., Rudan I. (2012). The association of dietary intake of purine-rich vegetables, sugar-sweetened beverages and dairy with plasma urate, in a cross-sectional study. PLoS One.

[76] Wang D.D., Sievenpiper J.L., de Souza R.J., Chiavaroli L., Ha V., Cozma A.I., Mirrahimi A., Yu M.E., Carleton A.J., Di Buono M. (2012). The effects of fructose intake on serum uric acid vary among controlled dietary trials. J. Nutr..

[77] Lonardo A., Loria P., Leonardi F., Borsatti A., Neri P., Pulvirenti M., Verrone A.M., Bagni A., Bertolotti M., Ganazzi D. (2002). Fasting insulin and uric acid levels but not indices of iron metabolism are independent predictors of non-alcoholic fatty liver disease. A case-control study. Dig. Liver Dis..

[78] Sartorio A., Del Col A., Agosti F., Mazzilli G., Bellentani S., Tiribelli C., Bedogni G. (2007). Predictors of non-alcoholic fatty liver disease in obese children. Eur. J. Clin. Nutr..

[79] Li Y., Xu C., Yu C., Xu L., Miao M. (2009). Association of serum uric acid level with non-alcoholic fatty liver disease: A cross-sectional study. J. Hepatol..

[80] Sertoglu E., Ercin C.N., Celebi G., Gurel H., Kayadibi H., Genc H., Kara M., Dogru T. (2014). The relationship of serum uric acid with non-alcoholic fatty liver disease. Clin. Biochem..

[81] Xie Y., Wang M., Zhang Y., Zhang S., Tan A., Gao Y., Liang Z., Shi D., Huang Z., Zhang H. (2013). Serum uric acid and non-alcoholic fatty liver disease in non-diabetic Chinese men. PLoS One.

[82] Sirota J.C., McFann K., Targher G., Johnson R.J., Chonchol M., Jalal D.I. (2013). Elevated serum uric acid levels are associated with non-alcoholic fatty liver disease independently of metabolic syndrome features in the United States: Liver ultrasound data from the National Health and Nutrition Examination Survey. Metabolism.

[83] Xu C., Yu C., Xu L., Miao M., Li Y. (2010). High serum uric acid increases the risk for nonalcoholic Fatty liver disease: A prospective observational study. PLoS One.

[84] Abdelmalek M.F., Suzuki A., Guy C., Unalp-Arida A., Colvin R., Johnson R.J., Diehl A.M. (2010). Increased fructose consumption is associated with fibrosis severity in patients with nonalcoholic fatty liver disease. Hepatology.

[85] Vos M.B., Colvin R., Belt P., Molleston J.P., Murray K.F., Rosenthal P., Schwimmer J.B., Tonascia J., Unalp A., Lavine J.E. (2012). Correlation of vitamin E, uric acid, and diet composition with histologic features of pediatric NAFLD. J. Pediatr. Gastroenterol. Nutr..

[86] Johnston R.D., Stephenson M.C., Crossland H., Cordon S.M., Palcidi E., Cox E.F., Taylor M.A., Aithal G.P., Macdonald I.A. (2013). No difference between high-fructose and high-glucose diets on liver triacylglycerol or biochemistry in healthy overweight men. Gastroenterology.

[87] Lanaspa M.A., Ishimoto T., Li N., Cicerchi C., Orlicky D.J., Ruzycki P., Rivard C., Inaba S., Roncal-Jimenez C.A., Bales E.S. (2013). Endogenous fructose production and metabolism in the liver contributes to the development of metabolic syndrome. Nat. Commun..

[88] Ishimoto T., Lanaspa M.A., Le M.T., Garcia G.E., Diggle C.P., Maclean P.S., Jackman M.R., Asipu A., Roncal-Jimenez C.A., Kosugi T. (2012). Opposing effects of fructokinase C and A isoforms on fructose-induced metabolic syndrome in mice. Proc. Natl. Acad. Sci. USA.

[89] Assy N., Nasser G., Kamayse I., Nseir W., Beniashvili Z., Djibre A., Grosovski M. (2008). Soft drink consumption linked with fatty liver in the absence of traditional risk factors. Can. J. Gastroenterol..

[90] Abid A., Taha O., Nseir W., Farah R., Grosovski M., Assy N. (2009). Soft drink consumption is associated with fatty liver disease independent of metabolic syndrome. J. Hepatol..

[91] Ouyang X., Cirillo P., Sautin Y., McCall S., Bruchette J.L., Diehl A.M., Johnson R.J., Abdelmalek M.F. (2008). Fructose consumption as a risk factor for non-alcoholic fatty liver disease. J. Hepatol..

[92] Zelber-Sagi S., Nitzan-Kaluski D., Goldsmith R., Webb M., Blendis L., Halpern Z., Oren R. (2007). Long term nutritional intake and the risk for non-alcoholic fatty liver disease (NAFLD): A population based study. J. Hepatol..

[93] Thuy S., Ladurner R., Volynets V., Wagner S., Strahl S., Konigsrainer A., Maier K.P., Bischoff S.C., Bergheim I. (2008). Nonalcoholic fatty liver disease in humans is associated with increased plasma endotoxin and plasminogen activator inhibitor 1 concentrations and with fructose intake. J. Nutr..

[94] Kanerva N., Sandboge S., Kaartinen N.E., Mannisto S., Eriksson J.G. (2014). Higher fructose intake is inversely associated with risk of nonalcoholic fatty liver disease in older Finnish adults. Am. J. Clin. Nutr..

[95] Mann C.J. (2003). Observational research methods. Research design II: Cohort, cross sectional, and case-control studies. Emerg. Med. J..

[96] Ngo Sock E.T., Le K.A., Ith M., Kreis R., Boesch C., Tappy L. (2010). Effects of a short-term overfeeding with fructose or glucose in healthy young males. Br. J. Nutr..

[97] Theytaz F., Noguchi Y., Egli L., Campos V., Buehler T., Hodson L., Patterson B.W., Nishikata N., Kreis R., Mittendorfer B. (2012). Effects of supplementation with essential amino acids on intrahepatic lipid concentrations during fructose overfeeding in humans. Am. J. Clin. Nutr..

[98] Sobrecases H., Le K.A., Bortolotti M., Schneiter P., Ith M., Kreis R., Boesch C., Tappy L. (2010). Effects of short-term overfeeding with fructose, fat and fructose plus fat on plasma and hepatic lipids in healthy men. Diabetes Metab..

[99] Lecoultre V., Egli L., Carrel G., Theytaz F., Kreis R., Schneiter P., Boss A., Zwygart K., Le K.A., Bortolotti M. (2013). Effects of fructose and glucose overfeeding on hepatic insulin sensitivity and intrahepatic lipids in healthy humans. Obesity.

[100] Scientific Advisory Committee on Nutrition (2012). Dietary Reference Values for Energy.

[101] Le K.A., Faeh D., Stettler R., Ith M., Kreis R., Vermathen P., Boesch C., Ravussin E., Tappy L. (2006). A 4-wk high-fructose diet alters lipid metabolism without affecting insulin sensitivity or ectopic lipids in healthy humans. Am. J. Clin. Nutr..

[102] Silbernagel G., Machann J., Unmuth S., Schick F., Stefan N., Haring H.U., Fritsche A. (2011). Effects of 4-week very-high-fructose/glucose diets on insulin sensitivity, visceral fat and intrahepatic lipids: An exploratory trial. Br. J. Nutr..

[103] Maersk M., Belza A., Stodkilde-Jorgensen H., Ringgaard S., Chabanova E., Thomsen H., Pedersen S.B., Astrup A., Richelsen B. (2012). Sucrose-sweetened beverages increase fat storage in the liver, muscle, and visceral fat depot: A 6-mo randomized intervention study. Am. J. Clin. Nutr..

[104] Bravo S., Lowndes J., Sinnett S., Yu Z., Rippe J. (2013). Consumption of sucrose and high-fructose corn syrup does not increase liver fat or ectopic fat deposition in muscles. Appl. Physiol. Nutr. Metab..

[105] Volynets V., Machann J., Kuper M.A., Maier I.B., Spruss A., Konigsrainer A., Bischoff S.C., Bergheim I. (2013). A moderate weight reduction through dietary intervention decreases hepatic fat content in patients with non-alcoholic fatty liver disease (NAFLD): A pilot study. Eur. J. Nutr..

[106] Jin R., Welsh J.A., Le N.A., Holzberg J., Sharma P., Martin D.R., Vos M.B. (2014). Dietary fructose reduction improves markers of cardiovascular disease risk in Hispanic-American adolescents with NAFLD. Nutrients.

[107] Koopman K.E., Caan M.W., Nederveen A.J., Pels A., Ackermans M.T., Fliers E., la Fleur S.E., Serlie M.J. (2014). Hypercaloric diets with increased meal frequency, but not meal size, increase intrahepatic triglycerides: A randomized controlled trial. Hepatology.

[108] Green C., Hodson L. (2014). The influence of dietary fat on liver fat accumulation. Nutrients.

[109] Heath R.B., Karpe F., Milne R.W., Burdge G.C., Wootton S.A., Frayn K.N. (2003). Selective partitioning of dietary fatty acids into the VLDL TG pool in the early postprandial period. J. Lipid Res..

[110] Chong M.F., Fielding B.A., Frayn K.N. (2007). Mechanisms for the acute effect of fructose on postprandial lipemia. Am. J. Clin. Nutr..

[111] Donnelly K.L., Smith C.I., Schwarzenberg S.J., Jessurun J., Boldt M.D., Parks E.J. (2005). Sources of fatty acids stored in liver and secreted via lipoproteins in patients with nonalcoholic fatty liver disease. J. Clin. Investig..

[112] Lambert J.E., Ryan E.A., Thomson A.B., Clandinin M.T. (2013). *De novo* lipogenesis and cholesterol synthesis in humans with long-standing type 1 diabetes are comparable to non-diabetic individuals. PLoS One.

[113] Hellerstein M.K., Christiansen M., Kaempfer S., Kletke C., Wu K., Reid J.S., Mulligan K., Hellerstein N.S., Shackleton C.H. (1991). Measurement of *de novo* hepatic lipogenesis in humans using stable isotopes. J. Clin. Investig..

[114] Wilke M.S., French M.A., Goh Y.K., Ryan E.A., Jones P.J., Clandinin M.T. (2009). Synthesis of specific fatty acids contributes to VLDL-triacylglycerol composition in humans with and without type 2 diabetes. Diabetologia.

[115] Hudgins L.C., Hellerstein M., Seidman C., Neese R., Diakun J., Hirsch J. (1996). Human fatty acid synthesis is stimulated by a eucaloric low fat, high carbohydrate diet. J. Clin. Investig..

[116] Peter A., Cegan A., Wagner S., Elcnerova M., Konigsrainer A., Konigsrainer I., Haring H.U., Schleicher E.D., Stefan N. (2011). Relationships between hepatic stearoyl-CoA desaturase-1 activity and mRNA expression with liver fat content in humans. Am. J. Physiol. Endocrinol. Metab..

[117] Kotronen A., Seppanen-Laakso T., Westerbacka J., Kiviluoto T., Arola J., Ruskeepaa A.L., Oresic M., Yki-Jarvinen H. (2009). Hepatic stearoyl-CoA desaturase (SCD)-1 activity and diacylglycerol but not ceramide concentrations are increased in the nonalcoholic human fatty liver. Diabetes.

[118] McNamara R.K., Magrisso I.J., Hofacer R., Jandacek R., Rider T., Tso P., Benoit S.C. (2012). Omega-3 fatty acid deficiency augments risperidone-induced hepatic steatosis in rats: Positive association with stearoyl-CoA desaturase. Pharmacol. Res..

[119] Jain M., Ngoy S., Sheth S.A., Swanson R.A., Rhee E.P., Liao R., Clish C.B., Mootha V.K., Nilsson R. (2014). A systematic survey of lipids across mouse tissues. Am. J. Physiol. Endocrinol. Metab..

[120] Musso G., Gambino R., Cassader M. (2013). Cholesterol metabolism and the pathogenesis of non-alcoholic steatohepatitis. Prog. Lipid Res..

[121] Debosch B.J., Chen Z., Saben J.L., Finck B.N., Moley K.H. (2014). Glucose transporter 8 (GLUT8) mediates fructose-induced de novo lipogenesis and macrosteatosis. J. Biol. Chem..

[122] Sun S.Z., Empie M.W. (2012). Fructose metabolism in humans—What isotopic tracer studies tell us. Nutr. Metab..

[123] Lambert J.E., Ramos-Roman M.A., Browning J.D., Parks E.J. (2014). Increased de novo lipogenesis is a distinct characteristic of individuals with nonalcoholic fatty liver disease. Gastroenterology.

[124] Diraison F., Moulin P., Beylot M. (2003). Contribution of hepatic de novo lipogenesis and reesterification of plasma non esterified fatty acids to plasma triglyceride synthesis during non-alcoholic fatty liver disease. Diabetes Metab..

[125] Faeh D., Minehira K., Schwarz J.M., Periasamy R., Park S., Tappy L. (2005). Effect of fructose overfeeding and fish oil administration on hepatic *de novo* lipogenesis and insulin sensitivity in healthy men. Diabetes.

[126] Fielding B.A., Umpleby A.M., Lovegrove J.A., Hodson L., Sharma S., Lanham-New S.A. (2015). Stable isotopes in Nutrition Research. Nutrition Research Methodologies.

